# Impact of COVID-19 infection in patients with inherited metabolic diseases: a National Multicenter Study from the French IMDs Healthcare Network for Rare Diseases

**DOI:** 10.1186/s13023-026-04230-8

**Published:** 2026-02-14

**Authors:** Claire Douillard, Aurélia Poujois, Nadia Belmatoug, Olivier Lidove, Vanessa Leguy-Seguin, Wladimir Mauhin, Magali Gorce, Aline Cano, Philippe Labrune, Karin Mazodier, Camille Wicker, François Maillot, Anaïs Brassier, Anne-Sophie Guemann, Dalila Habes, Marie-Thérèse Abi-Warde, Isabelle Redonnet-Vernhet, Dominique P. Germain, Christian Lavigne, Azza Khemiri, Karine Mention, Myriam Dao, Bénédicte Héron, Marc G. Berger, Pascale de Lonlay

**Affiliations:** 1 Service Endocrinologie-Diabétologie-Oncologie endocrinienne-Métabolisme, CHU Lille, Lille, France; 2 Département de Neurologie, Centre de référence de la maladie de Wilson et autres maladies rares liées au cuivre, Hôpital Fondation Adolphe de Rothschild, Paris, France; 3 Service de médecine Interne, Centre de Référence des Maladies Lysosomales, Hôpitaux Universitaires Paris Nord, Beaujon, AP-HP, Clichy France; 4 Service de Médecine Interne, Centre de Référence des Maladies Lysosomales, Groupe Hospitalier Diaconesses Croix Saint-Simon, Paris, France; 5 Service de Médecine interne et Immunologie Clinique, CHU, Dijon, France; 6 Centre de Référence des Maladies Héréditaires du Métabolisme, CHU Lille, Lille, France; 7 Centre de Référence des Maladies Héréditaires du Métabolisme, CHU Toulouse, Toulouse, France; 8 Centre de Référence des Maladies Héréditaires du Métabolisme- CHU La Timone Enfants, Marseille, France; 9 Centre de Référence des Maladies Héréditaires du Métabolisme Hépatique, Hôpital Antoine Béclère, APHP, Clamart, France; 10 Service de Médecine Interne, Centre de Référence des Maladies Héréditaires du Métabolisme, CHU Conception, Marseille, France; 11 Service de pédiatrie, Centre de Compétence des Maladies Héréditaires du Métabolisme, CHU Strasbourg, Strasbourg, France; 12 Médecine Interne, Centre Référence Maladies Héréditaires du Métabolisme, hôpital Bretonneau, CHU, Tours France; 13 Centre de Référence des Maladies Héréditaires du Métabolisme, Hôpital Universitaire Necker-enfants malades, AP-HP, Université Paris Cité, INSERM U1151, Institut Necker-Enfants Malades (INEM), Paris, France; 14 Service d’hépatologie pédiatrique, Hôpital Bicêtre, AP-HP, Le Kremlin- Bicêtre, France; 15 Service de Neurologie Pédiatrique, Centre de Compétence des Maladies Héréditaires du Métabolisme, CHU Strasbourg, Strasbourg, France; 16 Service d’Endocrinologie, Maladies Métaboliques et Nutrition, Centre de Compétence des Maladies Héréditaires du Métabolisme, Service de Biochimie, CHU Bordeaux, Bordeaux, France; 17 Service de génétique médicale, Centre de Référence pour la Maladie de Fabry et les maladies lysosomales, Hôpital Raymond Poincaré, AP-HP Paris Saclay, Garches, France; 18 Service de médecine interne et d’immunologie Clinique, CHU Angers, Angers, France; 19 Service de néphrologie, Hôpital Universitaire Necker-Enfants malades, AP- HP, Paris, France; 20 Service de Neuropédiatrie, Centre de Reference des Maladies Lysosomales, CH Armand Trousseau-La Roche Guyon, AP-HP, FHU I2-D2, Paris, France; 21 Filière G2m, Paris, France; 22 Centre de Compétence des Maladies Héréditaires du Métabolisme, Service Hématologie Clinique et service d’Hématologie Biologique, CHU Estaing, Université Clermont Auvergne, Clermont-Ferrand, France; 23 Filière G2m, MetabERN, Paris, France

**Keywords:** Inherited metabolic diseases, COVID-19, Metabolic decompensation, Rare diseases, Lysosomal disorders, Wilson disease, Phenylketonuria

## Abstract

**Background:**

The COVID-19 pandemic presented unique challenges for patients with inherited metabolic diseases (IMDs), particularly due to the risk of infection-related metabolic decompensation and disruptions to specialized care. We aimed to assess the impact of COVID-19 infection on the clinical course of patients with IMDs in a National Multicenter Study from the French IMDs Healthcare Network for Rare Diseases.

**Results:**

This national French study included 317 IMD patients (69 children and 248 adults) with symptomatic or asymptomatic COVID-19 infection between January 2020 and January 2023. Most COVID-19 cases were mild to moderate. The frequency of symptomatic COVID-19 was similar in adults and children (234/248 [94.3%] vs. 56/64 [87.5%], *p* = 0.09). Children experienced more frequently metabolic destabilization than adults during a COVID-19 infection (17/67 [25.4%] vs. 33/248 [13.3%], *p* = 0.03). Moreover, the proportion of children admitted to the ICU was higher than that of adult patients (5/69 [7.2%] vs. 4/248 [1.6%], *p* = 0.04). Temporary suspension or delay of IMD-specific treatment due to COVID-19 was rare, affecting 3/64 (4.7%) children and 13/229 (5.7%) adults. Severe COVID-19 outcomes were uncommon, with only one death in the adult cohort and five cases of long-term sequelae (1 child, 4 adults).

**Conclusions:**

COVID-19 was generally mild to moderate in IMD patients and caused metabolic decompensation or imbalance in a minority of cases, with only rare interruptions to disease-specific treatment. We observed that COVID-19 more frequently worsened the condition of children with IMD compared to adults in our cohort of patients.

**Supplementary Information:**

The online version contains supplementary material available at 10.1186/s13023-026-04230-8.

## Background

The coronavirus disease 2019 (COVID-19 or SARS-CoV-2) pandemic has placed significant strain on healthcare systems worldwide, presenting particularly acute challenges for patients with chronic rare conditions [1]. Most inherited metabolic diseases (IMDs) require complex, multidisciplinary care, involving coordinated acute and long-term management, as well as continuous monitoring to prevent disease decompensation. Both children and adults with IMDs were presumed to be vulnerable to severe outcomes from any infection, mainly due to their chronic metabolic instability and potentially fragile organ function, depending on the specific disease. These considerations initially raised concerns that COVID-19 could destabilize metabolic control in IMD patients and increase their risk of morbidity and mortality [2] .

Emerging evidence in 2020 indicated that the pandemic substantially disrupted the care of IMD patients worldwide. A global survey of 16 metabolic centers reported a 60–80% decline in IMD-related healthcare services during the first COVID-19 lockdown (March–May 2020) compared to 2019, highlighting a profound impact on routine diagnoses, treatments, and follow-up for this vulnerable population [3]. Similarly, the European Reference Network for Hereditary Metabolic Diseases (MetabERN) observed that most scheduled appointments and treatments for IMD patients were canceled, postponed, or reduced in the early phase of the pandemic [4]. Such interruptions are especially perilous for patients with IMDs, who require continuous follow-up and ongoing management to prevent metabolic decompensations and disease progression.

But data on the direct outcomes of COVID-19 infection in patients with IMDs remained scarce. One year into the pandemic, a follow-up MetabERN survey documented 452 confirmed COVID-19 cases among approximatively 26,300 IMD patients, corresponding to a cumulative prevalence of about 1.7% [5]. Most IMD cases – both pediatric and adult – were asymptomatic or mild, with very few acute deaths attributable to COVID-19. However, some severe cases occurred, including fatal outcomes in a subset of pediatric IMD patients. The long-term consequences on metabolic control and organ function remain unknown and are a concern. The reliance on surveys limits the availability of detailed clinical data, underscoring the need for further research to better understand COVID-19’s true impact on IMD patients’ health and long-term outcomes. Overall, the pandemic’s specific effects on patients with IMD are only partially understood, with limited robust outcome data.

To address these knowledge gaps, the present study (“COVID-MHM”) was initiated to investigate the impact of COVID-19 infection on patients with IMDs. COVID-MHM is a multicenter observational study conducted between 2021 and 2023 within the French IMDs Healthcare Network for Rare Diseases, known as “Filière G2m. The primary objective was to evaluate how COVID-19 infection affected both the clinical course of IMDs and their management. By collecting detailed data on pediatric and adult IMD patients with COVID-19, including metabolic decompensations or metabolic imbalances, treatment modifications, and care delays, this study aims to provide a comprehensive assessment of COVID-19 outcomes in the IMD population.

## Methods

### Study design and objectives

The COVID-MHM study (NCT04645498; date of registration : 2020-11-27) was a multicenter, ambispective, observational cohort study conducted through the French Inherited Metabolic Diseases Healthcare Network (Centres de Référence des Maladies Héréditaires du Métabolisme, CRMR-MHM, Centres de Compétence des Maladies Héréditaires du Métabolisme, CCMR-MHM). Its primary objective was to assess the impact of COVID-19 infection on the clinical course of patients with IMDs, specifically on metabolic imbalance, metabolic decompensation, worsening of IMD-related symptoms, treatment disruptions, and infection-related outcomes.

### Study population and inclusion criteria

Patients were included from 20 expert centers across France. Eligible participants were pediatric (< 18 years) or adult (≥ 18 years) patients with a confirmed diagnosis of IMD, who experienced a SARS-CoV-2 infection (documented by PCR, antigenic test, or serology before vaccination) between January 2020 and January 2023. Patients with both asymptomatic and symptomatic COVID-19 infection were included. Written informed consent was obtained from all participants or their legal guardians, in accordance with French ethical standards (ethical approval: 2020-A02886-33).

### Data collection

Standardized electronic case report forms were used by participating clinicians to collect detailed data in a harmonized format. Collected variables included:

- **Demographics and disease characteristics**: age, sex, pediatric or adult status.
- **Specific IMD diagnosis:**
  - Intoxication disorders (e.g. aminoacidopathies, organic acidurias, metal accumulation (e.g. Wilson disease), hepatic porphyria).
  - Primarily energy metabolism disorders (e.g. fatty acid oxidation disorders (FAOD), mitochondrial diseases, glycogen storage diseases (GSD), congenital hyperinsulinism).
  - Complex molecules disorders [e.g. lysosomal disorders (LDs), congenital disorders of glycosylation (CDG)].
- **COVID-19-related variables**: infection date; symptoms (fever, myalgia, cold-like symptoms, cough, shortness of breath, diarrhea, sudden severe fatigue, loss of taste and smell, atypical presentations); severity (pneumonia, ARDS-acute respiratory distress syndrome, the need for hospitalization or ICU admission); COVID-19 outcome (recovery, sequelae, death).
- **IMD-specific clinical consequences**: the impact of COVID-19 on IMD was defined by an expert clinician in IMD, according to the following criteria:
  1. No effect on IMD.
  2. Impact on IMD defined by either:
    Clinical symptoms and/or biological markers (see Supplemental Data 1) of acute metabolic decompensation and/or abnormal biochemical markers indicating disease imbalance,
    Worsening of IMD-related symptoms or complications, or destabilization of metabolic disease,
    Need for emergency interventions (e.g. intensified dietary and/or pharmacologic measures and/or hospitalization).
- **Occurrence of treatment disruptions**: assessment of COVID-19’s impact on IMD treatment, including temporary discontinuation of disease-specific therapies (e.g., enzyme replacement, chelators or zinc for Wilson Disease, medical diet support), postponement or cancellation of scheduled care, or shift to home-based alternatives.
- **Medium- and long-term outcomes**: presence of residual symptoms or new complications following COVID-19, and any death attributed to metabolic or infectious complications.
- **Presence of comorbidities**: defined as having at least one of the following conditions: diabetes, obesity, active or former smoking, hypertension, heart disease, pulmonary disorders, renal insufficiency, or immunodeficiency.

### Statistical analysis

Descriptive statistics were applied to summarize patient characteristics, COVID-19 severity, and IMD-related outcomes. Categorical data were compared using chi-squared or Fisher’s exact tests, while continuous variables were analyzed using Student’s *t*-test or the Mann–Whitney *U* test, as appropriate. A *p*-value < 0.05 was considered statistically significant. Analyses were performed using GraphPad Prism 10.5.0.

## Results

### Characteristics of the study population

A total of 317 patients with IMDs and COVID-19 infection were included from 20 French expert centers between January 2020 and January 2023: 50 patients in 2020, 77 in 2021, and 151 in 2022 (39 missing data regarding the year of infection). Among them, 69 were children (< 18 years, 21.8%) and 248 were adults (≥ 18 years, 78.2%).

**Fig. 1:**
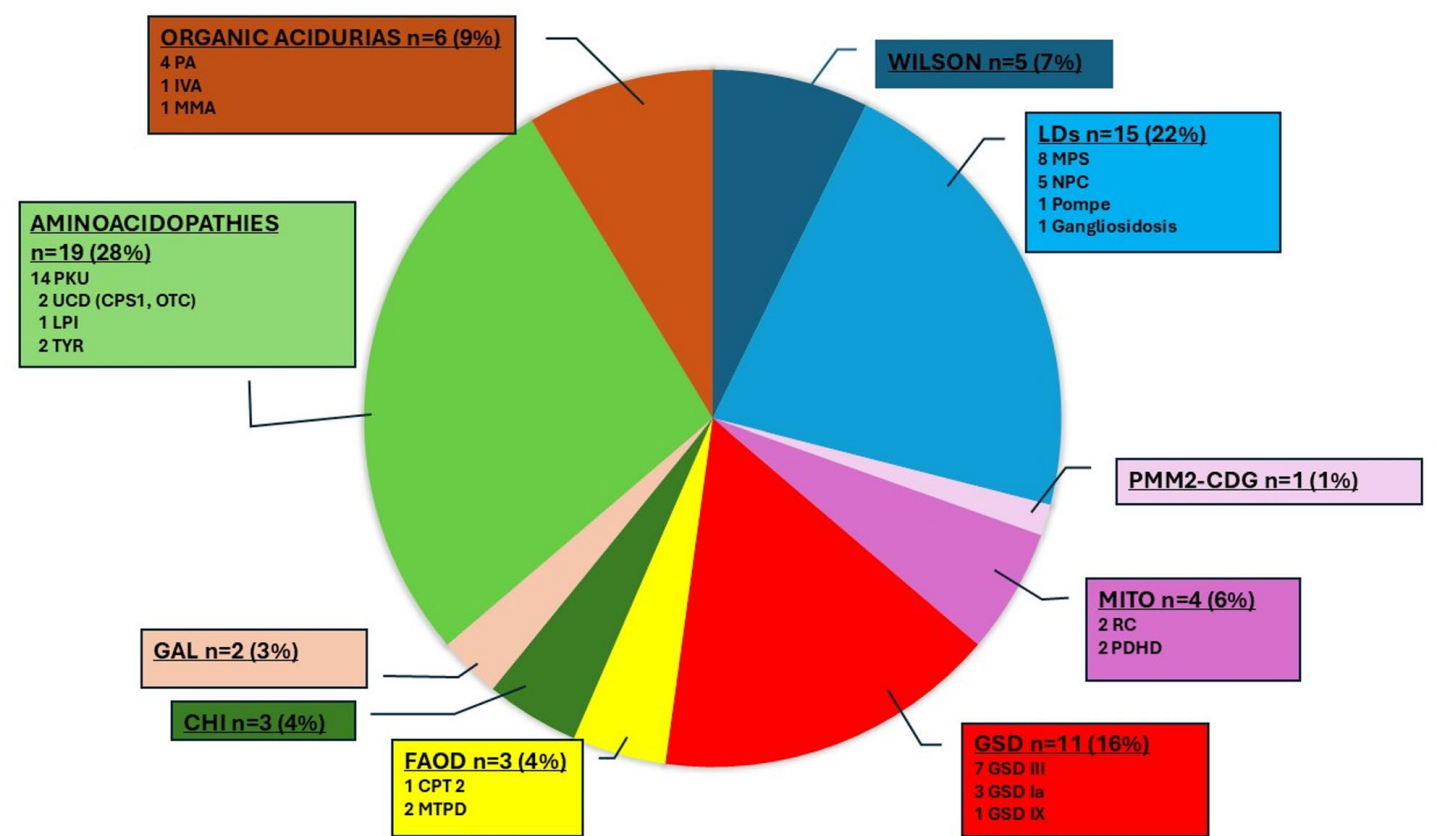
Distribution of the different IMD in Children affected by COVID-19 (*n* = 69). CHI: Congenital hyperinsulinism; CPS1: Carbamylphosphate Synthase 1; CPT 2: Carnitine palmitoyltransferase 2; GAL: Galactosemia; GSD: Glycogen storage disease; IVA: Isovaleric aciduria; LPI: Lysinuric Protein Intolerance; LDs: Lysosomal disorders; MITO: Mitochondrial diseases; MMA: Methylmalonic aciduria; MPS: Mucopolysaccharidoses; MTPD: Mitochondrial trifunctional protein deficiency; NPC: Niemann-Pick C disease; OTC: Ornithine Transcarbamylase; PA: Propionic aciduria; PDHD: Pyruvate dehydrogenase deficiency; PKU: Phenylketonuria; PMM2-CDG: Phosphomannomutase deficiency - Congenital disorders of glycosylation; Pompe: Pompe disease; RC: Respiratory Chain deficiency; TYR: Tyrosinemia type 1; UCD: Urea cycle disorders; Wilson: Wilson disease

**The pediatric group** (*n* = 69) had a mean age of 10.2 ± 4.6 years (range 0.8–17.9), with an equal sex distribution (34 males, 35 females). The distribution of the different IMDs is detailed in Table 1. The most frequently affected disease groups were: (1) aminoacidopathies, (2) LDs, and (3) GSD. The most common IMDs were phenylketonuria (PKU, *n* = 14), mucopolysaccharidoses (MPS, *n* = 8), GSD III (*n* = 7), see Fig. 1. Six children (8.7%) had at least one comorbidity.

**Table 1:** Characteristics of the population and impact of COVID-19 infection

	*n*	children	*n*	adults	*p*
Number of patients (*n* = 317)		69		248	
Age	69	10.2 ± 4.6	248	39.9 ± 14.7	< 0.0001
Sex (males/females)	69	34/35	245	103/142	0.35
Comorbidities	69	6 (8.7%)	248	101 (40.7%)	< 0.0001
Symptomatic COVID-19	64	56 (87.5%)	248	234 (94.3%)	0.09
ICU admission	69	5 (7.2%)	248	4 (1.6%)	0.04
Due to IMD destabilization		3 (MITO, FAOD, GSD III)		0	
Due to COVID-19 severity		2 (PMM2-CDG, MPS II)		4 (2 FABRY, 2 ASMD)	
IMD destabilization	67	17 (25.4%)	248	33 (13.3%)	0.027

**Fig. 2:**
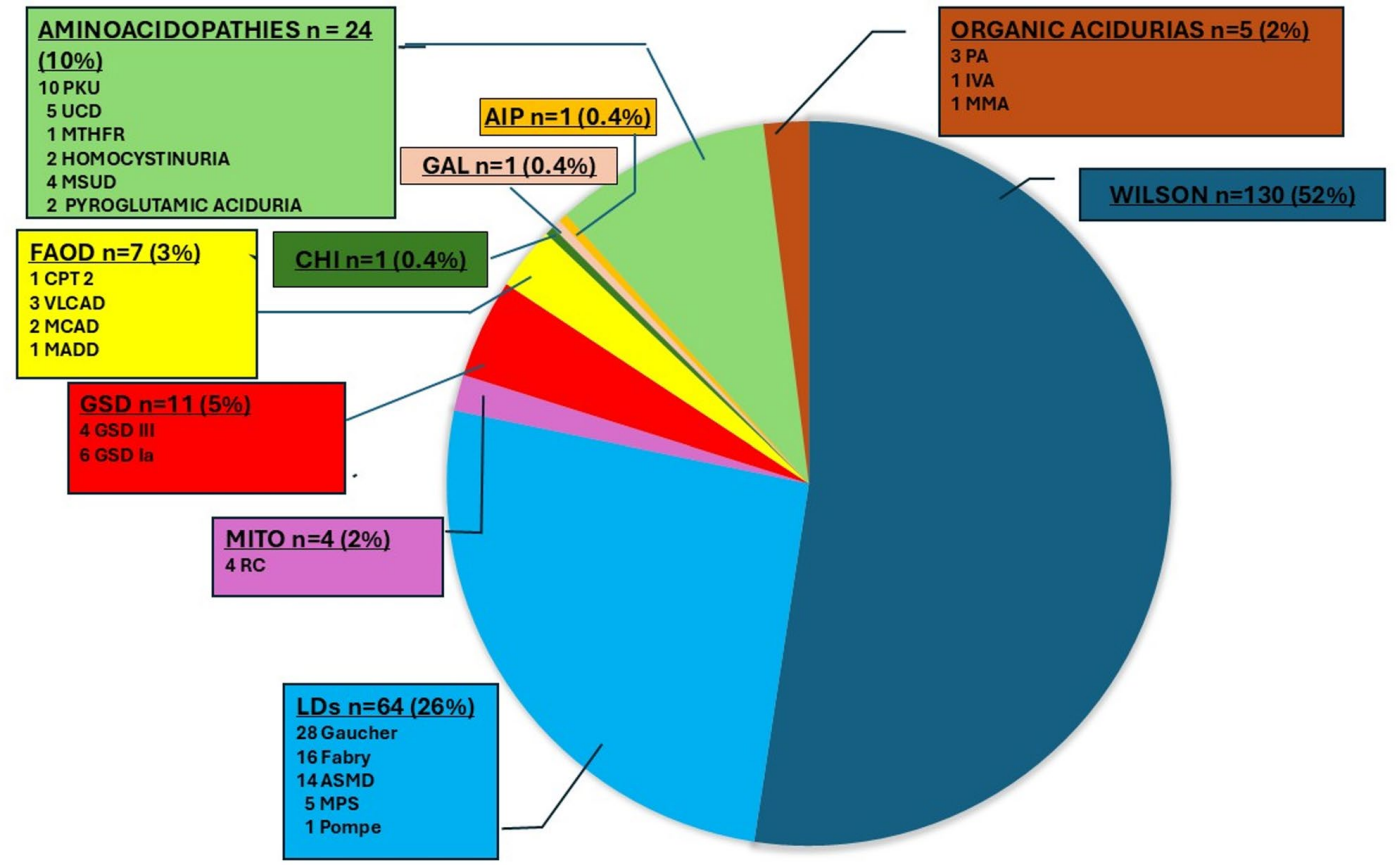
Distribution of the different IMD in Adults affected by COVID-19 (*n* = 248). AIP: Acute intermittent porphyria; ASMD: Acid sphingomyelinase deficiency; CHI: Congenital hyperinsulinism; CPT 2: Carnitine palmitoyltransferase 2; FAOD: Fatty acid oxidation disorders; Fabry: Fabry disease; GAL: Galactosemia; GSD: Glycogen storage disease; Gaucher: Gaucher disease; IVA: Isovaleric aciduria; LDs: Lysosomal disorders; MADD: Multiple acyl-CoA dehydrogenase deficiency; MCAD: Medium-chain acyl-CoA dehydrogenase deficiency; MITO: Mitochondrial diseases; MMA: Methylmalonic aciduria; MPS: Mucopolysaccharidoses; MTHFR: Methylene-tetrahydrofolate reductase; MSUD: Maple syrup urine disease; NPC: Niemann-Pick C disease; PA: Propionic aciduria; PKU: Phenylketonuria; Pompe: Pompe disease; RC: Respiratory Chain deficiency; TYR: Tyrosinemia type 1; UCD: Urea cycle disorders (5 UCD including 4 OTC and 1 UCD unspecified); VLCAD: Very long-chain acyl-CoA dehydrogenase deficiency; Wilson: Wilson disease

**The adult group** (*n* = 248) had a mean age of 39.9 ± 14.7 years (range 18.1–93.1). Among the 245 patients with recorded sex, 103 were men and 142 women. The distribution of diseases is detailed in Table 1. The most frequently affected disease groups were: (1) Wilson disease, (2) LDs, and (3) aminoacidopathies. The most common IMDs were Wilson disease (*n* = 130, 52%), followed by Gaucher disease (*n* = 28), Fabry disease (*n* = 16) and acid sphingomyelinase deficiency (ASMD, *n* = 14), see Fig. 2. One hundred and one adult patients (40.7%) had at least one comorbidity.

Compared to children, adult patients with IMD had a similar sex ratio but a higher prevalence of comorbidities (101/248 (40.7%) vs. 6/69 (8.7%), *p* < 0.0001).

### Clinical presentation of COVID-19

290 patients (92.9% of the cohort) presented with symptomatic COVID-19 infection, while 22 (7.1%) were asymptomatic (data were missing in 5 children). Among children, 56 (87.5%) developed symptomatic COVID-19, while 8 (12.5%) were asymptomatic. In adults, 234 out of 248 (94.3%) had symptomatic infections, while 14 patients (5.6%) were asymptomatic. The proportion of symptomatic patients was similar between adults and children (94.3% vs. 87.5%, *p* = 0.09).

**In children**, 13/69 (18.8%) patients were hospitalized, including 2 (2.9%) primarily due to COVID-19 severity, 9 (13.0%) due to impact of COVID-19 on IMD, and 2 (2.9%) for unspecified reasons. A total of 5 children (7.2%) required ICU admission.

**In adults**, 19/248 (7.6%) were hospitalized: 6 (2.4%) primarily for COVID-19, 10 (4.0%) due to the impact of COVID-19 on IMD, and 3 (1.2%) for unspecified reasons. Among the 6 adults hospitalized primarily for COVID-19 (2 with ASMD, 2 with Fabry disease, 1 galactosemia, 1 with Gaucher disease), 4 required ICU (including 2 with Fabry disease and 2 with ASMD), and these were the only adults requiring ICU. One female patient with Fabry disease and comorbidities died at age 75. The overall mortality rate was 0.3%, and among adults, it was 0.4%.

The proportion of children admitted to ICU was higher than that of adults (5/69 (7.2%) vs. 4/248 (1.6%), *p* = 0.04).

### Impact COVID-19 on IMD

Data on the stability of metabolic disease during or following COVID-19 were available for 67 children and 248 adults.

**Fig. 3:**
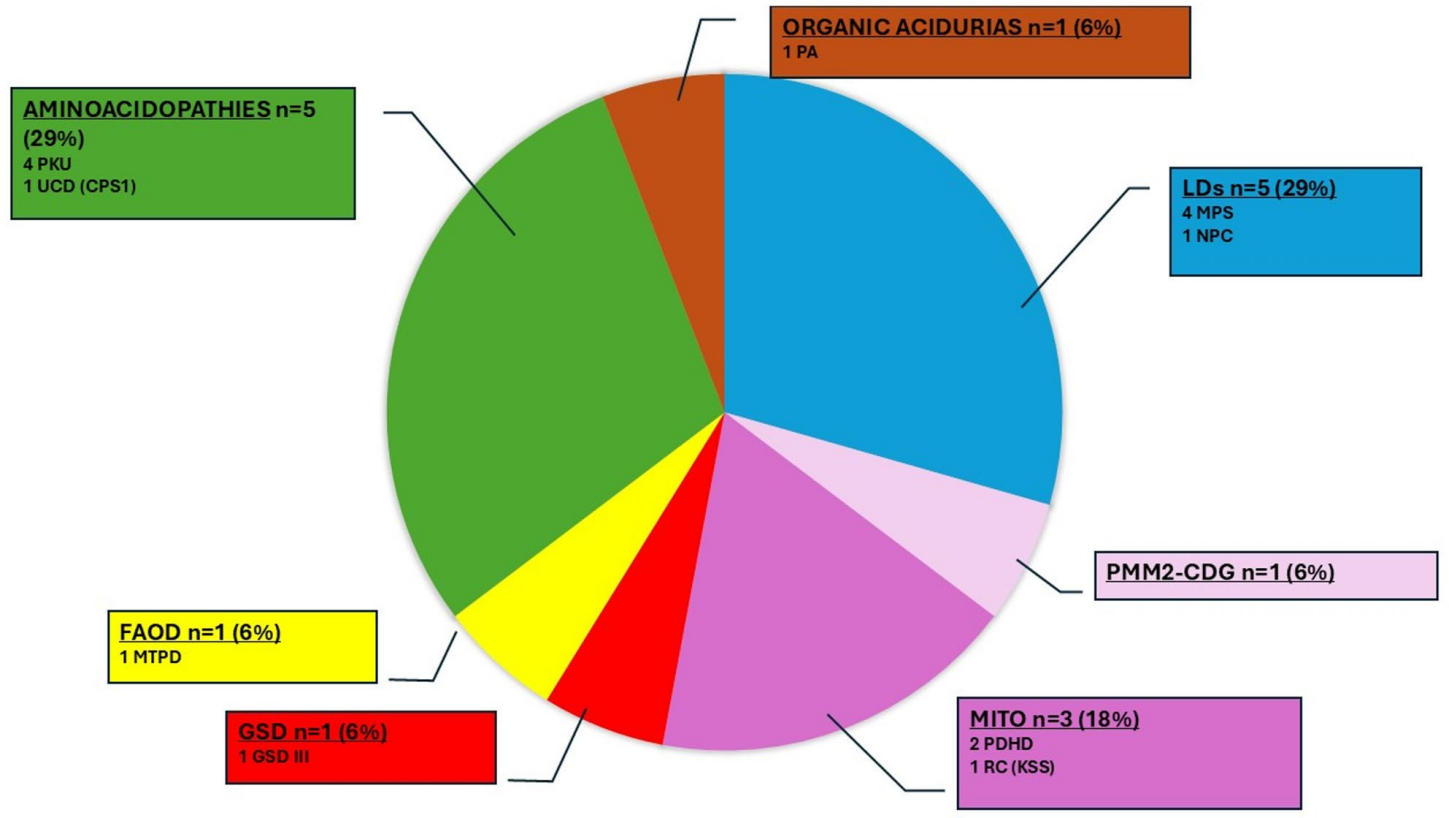
Distribution of the different IMD impacted by COVID 19 in children (*n* = 17). CPS1: Carbamylphosphate Synthase 1; FAOD: Fatty acid oxidation disorders; GSD: Glycogen storage disease; KSS: Kearns-Sayre syndrome; LDs: Lysosomal disorders; MITO: Mitochondrial diseases; MPS: Mucopolysaccharidoses; MTPD: Mitochondrial trifunctional protein deficiency; NPC: Niemann-Pick C disease; PA: Propionic aciduria; PDHD: Pyruvate dehydrogenase; PKU: Phenylketonuria; PMM2-CDG: Phosphomannomutase deficiency - Congenital disorders of glycosylation; RC: Respiratory Chain deficiency; UCD: Urea cycle disorders

**In children**, 17 patients out of 67 (25.4%) experienced an impact of COVID-19 on IMD (see Fig. 3). Among them, 11 were hospitalized: 2 primarily for COVID-19 severity (1 with MPS II and 1 with Phosphomannomutase deficiency - Congenital disorders of glycosylation (PMM2-CDG), both requiring ICU) which subsequently led to negative consequences on the IMD itself, and 9 primarily due to an impact of COVID-19 on IMD, including 3 with MPS (2 MPS IV, 1 MPS III), 2 with mitochondrial diseases [Kearns-Sayre syndrome (KSS), pyruvate dehydrogenase deficiency (PDHD)], 1 with PCU, 1 with urea cycle disorder (UCD : Carbamylphosphate Synthase 1 (CPS1) deficiency), 1 with GSD III, and 1 with FAOD (Mitochondrial trifunctional protein deficiency (MTPD)). Among these 9 patients hospitalized for an impact of COVID-19 on IMD, 3 required ICU care: 1 with mitochondrial disease (KSS), 1 with GSD III, and 1 with FAOD (MTPD). Finally, six patients had an impact of COVID-19 on IMD but without hospitalization, including 1 with organic aciduria (propionic acidemia), 3 with PKU, 1 with Niemann-Pick C, and 1 with mitochondrial disease (PDHD).

In total, five children required ICU care: 2 due to severe COVID-19 (patients with MPS II and PMM2-CDG) leading to negative consequences on the IMD itself, and 3 due to metabolic decompensation of energy deficiency disorders (mitochondrial disease, FAOD, GSD III).

**Fig. 4:**
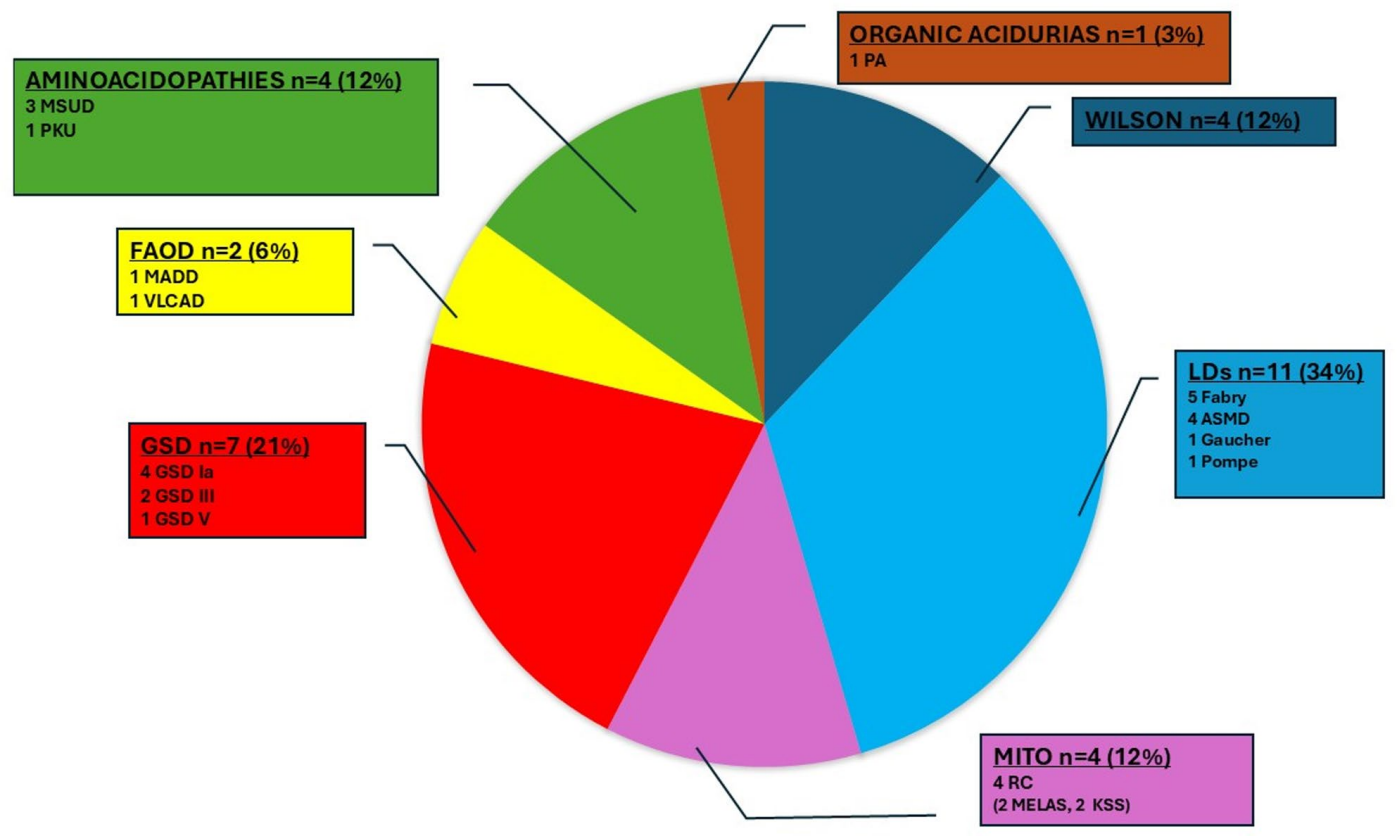
Distribution of the different IMD impacted by COVID 19 in adults (*n* = 33). ASMD: Acid sphingomyelinase deficiency; FAOD: Fatty acid oxidation disorders; Fabry: Fabry disease; GSD: Glycogen storage disease; Gaucher: Gaucher disease; KSS: Kearns-Sayre syndrome; LDs: Lysosomal disorders; MADD: Multiple acyl-CoA dehydrogenase deficiency; MELAS: Mitochondrial Encephalomyopathy Lactic Acidosis Stroke-like episodes; MITO: Mitochondrial diseases; MSUD: Maple syrup urine disease; PA: Propionic aciduria; PKU: Phenylketonuria; Pompe: Pompe disease; RC: Respiratory Chain deficiency; VLCAD: Very long-chain acyl-CoA dehydrogenase deficiency; Wilson: Wilson disease

**In adults**, 33 out of 248 patients (13.3%) experienced an impact of COVID-19 on IMD (see Fig. 4). Among them, 14 were hospitalized: four were admitted to the ICU primarily due to COVID-19 severity, leading to metabolic destabilization (2 with Fabry disease, 2 with ASMD), and 10 were hospitalized mainly for an impact of COVID-19 on IMD (1 with Very long-chain acyl-CoA dehydrogenase deficiency (VLCAD), 1 with Maple syrup urine disease (MSUD), 3 with GSD Ia, 1 with Wilson disease, 2 with Fabry disease, 1 with Gaucher disease, and 1 with ASMD), none of whom required ICU care. Finally, 19 patients experienced an impact of COVID-19 on IMD but without hospitalization, including 1 with PKU, 1 with organic aciduria (propionic acidemia), 4 with mitochondrial disease (respiratory chain deficiency: 2 MELAS, 2 KSS), 1 with Multiple acyl-CoA dehydrogenase deficiency (MADD), 4 with GSD (2 GSD III, 1 GSD Ia, 1 GSD V), 2 with MSUD, 1 with Pompe disease, 3 with Wilson disease, 1 with ASMD, and 1 with Fabry disease. In adults, the frequency of pre-existing common comorbidities (e.g., hypertension, diabetes, obesity) was not higher in patients with an imbalance or metabolic decompensation during COVID-19 than in patients without (13/33 [39.4%] vs. 88/215 [40.9%], *p* > 0.99).

In total, ICU admission in the four adult patients was primarily due to severe COVID-19, affecting individuals with LDs.

Interestingly, the frequency of metabolic destabilization was higher in children than adults (25.4% vs. 13.3%, *p* = 0.03, chi-squared test).

As vaccination began in France in January 2021, we compared the number of patients hospitalized or admitted to the ICU in 2020 versus 2021–2022 in children and adults (Supplemental Table 1). Although a decrease was observed in 2021–2022 compared with 2020, this difference was not statistically significant.

### Treatment modifications and long-term outcomes

Data on treatment modifications were missing in 5/69 (7.2%) children and 19/248 (7.6%) adults. Among patients with available data, COVID-19 caused a temporary suspension or delay of IMD-specific treatment in 3/64 (4.7%) children and 13/229 (5.7%) adults (p = NS).

In children, interruptions concerned enzyme replacement therapy (ERT), including 1 patient with Pompe disease (4 weeks) and 2 patients with MPS IV (8 weeks).

In adults, treatment was interrupted in 9 LDs patients (interruption of ERT in 5 Gaucher disease, 2 ASMD, 2 Fabry disease) and 4 patients with Wilson disease due to chelating agent interruption. One adult with ASMD permanently discontinued treatment while the other adult patients paused treatment for an average of 4.2 ± 3.0 weeks.

Long-term sequelae attributable to COVID-19 were reported in five cases: one child with MPS II and four adults with Wilson disease, GSD III, ASMD, and organic aciduria. These sequelae included persistent fatigue, organ dysfunction, or sustained biochemical abnormalities, although detailed clinical descriptions were variably available.

## Discussion

In this study, we assessed the severity of COVID-19 and its impact on disease control and treatment in a cohort of 317 patients with IMD from the French IMDs Healthcare Network for Rare Diseases.

### Severity of COVID-19 in patients with IMDs

First, in our cohort, patients with IMD did not appear to suffer disproportionately severe COVID-19 outcomes compared to the general population, and the COVID-19–related mortality rate was low as 1 patient among 317 died. Symptomatic cases were more frequent than asymptomatic ones among the patients included in our study, with no significant difference between adult and pediatric populations. However, this result must be nuanced as symptomatic patients were more likely to be diagnosed and included in our study than asymptomatic patients. In Lampe et al. study, the majority of IMD patients who contracted COVID-19 were either asymptomatic or experienced only mild illness: early surveys in 2020 recorded very few IMD patients with COVID-19, and nearly all had mild symptoms or none at all [2]. As the pandemic progressed and more cases were reported, most metabolic centers continued to observe that the vast majority of both pediatric and adult IMD cases were mild or moderate [5, 6]. Severe COVID-19 – defined by pneumonia requiring hospitalization or intensive care – was observed in a minority of cases, and COVID-19-related mortality among IMD patients remained low overall [4, 7].

In one study involving 223 patients, including 131 children, COVID-19 severity was generally comparable to that of the general population, without a disproportionate risk of acute metabolic decompensation compared to other infections. Children with LSD showed an increased risk of severe COVID-19, probably related to their multisystem involvement [6]. In our study, the proportion of children who required hospitalization was higher than that of adult patients, even though they had fewer comorbidities. Notably, the proportion of children admitted to the ICU was also higher than that of adult patients. Importantly, in children, ICU admission was primarily due to metabolic decompensation of the underlying energy metabolism disorder in 3 out of 5 cases. In contrast, in adults, ICU admission was always related to the severity of COVID-19 itself - often followed by destabilization of metabolic disease - particularly in patients with LDs, including one death in a 75-year-old patient with Fabry disease and associated comorbidities. Although we acknowledge that we cannot draw firm conclusions due to the low mortality and low number of patients admitted to ICU in our cohort, our data align with European data, in which some metabolic centers (~ 15% in one survey) reported at least one COVID-19 related fatality among their adult IMD patients, while nearly no pediatric deaths were noted [4, 5].Of note, children very rarely developed multisystem inflammatory syndrome (MIS-C) which was not observed in our cohort [8]. Finally, we were not able to estimate the incidence of COVID-19 among the active IMD patient cohort in our country, but it had been estimated at the European level in 2020: initial survey results suggested a relatively low incidence of COVID-19 among IMD patients during the first wave. For example, between March and April 2020, the estimated COVID-19 incidence in the MetabERN cohort was 72.9 per 100,000, lower than the ~ 117 per 100,000 observed in the general European population at the time [2]. The most likely explanation put forward was the mobilization of healthcare professionals to provide strict protective guidelines to patients with an IMD.

### Pediatric vs. adult outcomes and frequency of IMD decompensation

In our study, the frequency of metabolic decompensation following COVID-9 infection was higher in children than adults. Three out 5 children admitted in ICU had primarily energy metabolism disorders while all adult patients admitted in ICU had lysosomal disorders The prevalence of comorbidities was five times higher in adult patients than in children in our study, which likely contributes to the increased frequency of severe COVID-19 [9]. Conversely, in our cohort, the presence of comorbidities in adults did not appear to influence the frequency of metabolic imbalance, IMD destabilization, or IMD-decompensation due to COVID-19.

No death was observed among children. This suggests that medical management and emergency protocols for IMDs helped mitigate the risk of metabolic decompensation during COVID-19, especially in younger patients [10]. Close coordination within the metabolic care community, including the rapid dissemination of information by the National Healthcare Network for IMDs (G2m) and the use of telemedicine by each center likely helped protect patients. In addition, the reduced infection rate has been attributed to strict shielding measures and preventive behaviors adopted by IMD patients and their caregivers (social distancing, mask-wearing, and hygiene measures) [4].

### Disease-specific insights

#### Lysosomal disorders (LDs)

Patients with lysosomal storage diseases, such as Gaucher disease, Fabry disease, and Pompe disease, were initially thought to be at high risk for severe COVID-19 due to underlying multisystem involvement (e.g. cardiomyopathy or pulmonary dysfunction in some LDs). However, in practice, reported COVID-19 outcomes in patients with LDs have been mostly reassuring. For instance, a case series of late-onset Pompe disease (a LD causing respiratory muscle weakness) found that all four monitored patients had only mild to moderate COVID-19 illness, and none required hospitalization [11]. In a MetabERN survey, LDs were the most frequently IMD affected by COVID-19 in adults, with a favorable outcome [5]. Nevertheless, in our study, adult patients admitted to the ICU presented with LDs, including one death in a patient with Fabry disease and associated comorbidities.

**Fabry disease** shares many pathophysiological pathways with COVID-19, which could worsen outcomes, although certain Fabry disease specific factors may modulate infection severity. Both conditions are characterized by endothelial dysfunction and vasculopathy with Angiotensin II–mediated endothelial dysfunction [12, 13], . Direct endothelial infection by SARS-CoV-2, resulting in “endotheliitis,” may therefore affect an endothelium already compromised by Fabry disease related nitric oxide (NO) deficiency, excess reactive oxygen species, and overexpression of adhesion molecules that promote leukocyte adhesion [14]. Likewise, both Fabry disease and acute COVID-19 stimulate inflammatory cytokines release and a prothrombotic state [15]. These overlapping mechanisms may converge and amplify organ damage in the heart, brain, and kidneys — organs already affected in Fabry disease and known to be risk factors for severe COVID-19 outcomes [16, 17]. Conversely, patients with less advanced Fabry disease (with minimal organ fibrosis) do not appear to be inherently more vulnerable to severe COVID-19 than the general population.

Interestingly, emerging data suggest that lysosomal dysfunction in Fabry disease may even attenuate SARS-CoV-2 propagation and severity: glycosphingolipid accumulation can raise endolysosomal pH, impair ACE2 glycosylation and cathepsin L activity, thereby hindering efficient viral entry and replication [18]. Thus, the unique cellular environment in Fabry disease (and its treatment with RAS blockers or enzyme replacement therapy) could in some cases reduce viral infectivity or excessive immune responses. However, patients with Fabry disease and renal graft are susceptible to develop a weak response to COVID-19 vaccination highlighting the importance of maintaining barrier protection measures. Vaccination of family members should be encouraged to lower the risk of viral transmission to immunocompromised, transplanted patients [19].

Regarding **Gaucher disease**, studies have reported a milder-than-expected course in affected patients. This may reflect a baseline immune profile that prevents the uncontrolled acute cytokine storm seen in severe COVID-19. In one report, Gaucher disease patients infected with SARS-CoV-2 showed only mild elevations in pro-inflammatory cytokines, which normalized quickly during convalescence [20]. Another factor is therapy: many patients receive substrate reduction therapy, and inhibitors of glucosylceramide synthase - the enzyme upstream of glycolipid accumulation - have been shown to inhibit SARS-CoV-2 replication in vitro [21]. This therapeutic modulation of glycosphingolipid levels could theoretically interfere with viral entry or assembly.

Finally, a significant issue in this group was the interruption of therapy during pandemic lockdowns. Many LDs patients rely on regular intravenous ERT, typically administered in hospitals. During the first wave of the pandemic, up to ~ 49% of European LDs patients experienced disruptions in their ERT infusions due to hospital service limitations [2]. Missed or delayed infusions raised concerns about disease control (for example, stability of cardiac or neurologic status in Pompe or Fabry disease) and caused anxiety among patients. In response, several centers transitioned eligible patients to home-based infusions or spaced out dosing intervals to ensure continuity of care [2]. Overall, COVID-19 infection severity in LDs patients was generally mild, and the main impact of the pandemic on this group was organizational – maintaining treatment schedules and monitoring in the face of lockdowns – rather than direct viral complications.

**Wilson disease** often causes chronic liver disease and even neuropsychiatric symptoms (European Association for the Study of the Liver, EASL-ERN Clinical Practice Guidelines on Wilson’s disease) [22]. Since chronic liver disease increases the risk for severe COVID-19, Wilson disease patients – especially those with cirrhosis – were considered as high-risk. Neurological patients with dysphagia and recurrent respiratory infections were also at increased risk of pulmonary decompensation. However, most stable Wilson disease patients who contracted the virus did not experience more severe illness than expected based on their liver condition. Rare severe cases, like a 13-year-old boy with advanced liver disease who developed fatal COVID-19-induced multisystem inflammatory syndrome (MIS-C) with acute liver failure and multi-organ failure, highlight that severe COVID-19 can be dangerous for Wilson disease [23]. This emphasizes the importance of prompt anti-inflammatory treatment and consideration of early transplantation in such scenarios. Apart from such extreme cases, our cohort’s experience aligns with international observations: with careful maintenance of their anti-copper therapy and monitoring, most Wilson disease patients navigated COVID-19 without major incident [24]. The pandemic’s main impact on this group was on healthcare access and ongoing management rather than acute infection outcomes. A survey from an Indian center highlighted significant challenges for Wilson disease patients during lockdown: one-third of patients struggled to obtain their chelation medications, routine monitoring was disrupted, and about 22% experienced worsening liver or neurological symptoms after stopping therapy [25]. Only 2 out of 45 patients in that study used telemedicine, as many lacked the resources or awareness to do so [25]. Nonetheless, the pandemic highlighted the need for reliable access to medications and remote care for Wilson disease patients, to prevent interruptions that could precipitate decompensation.

**Phenylketonuria** special features: As a metabolic disorder managed primarily through diet, PKU presents a somewhat different scenario. PKU patients are generally healthy if their phenylalanine-restricted diet is well controlled, and they were therefore not considered to have intrinsic susceptibility to severe COVID-19. Our findings and published data confirm that PKU patients did not experience any direct worsening of COVID-19 outcomes [4].The challenges faced by PKU patients during the pandemic were mainly nutritional and psychological. Lockdowns disrupted supply chains and daily routines, raising concerns about access to specialized low-protein foods and medical formulas. Many PKU patients reported anxiety about potential shortages of metabolic foods [26]. Despite these concerns, clinics reported that metabolic control in PKU patients remained stable throughout the pandemic [26]. This suggests that dietary management programs adapted effectively. In our study, COVID-19 led to an imbalance in some PKU patients, even requiring hospitalization in one child to restore metabolic control, although there was no risk of acute decompensation.

#### Other aminoacidopathies and organic acidurias

Inborn errors of branched-chain amino acid metabolism (MSUD), UCD, and organic acid metabolism (e.g., propionic aciduria, methylmalonic aciduria) were a major concern during the COVID-19 pandemic, since these “intoxication-type” IMDs are prone to acute metabolic decompensation during infections.

Surprisingly, both our data and international surveys show that many patients with aminoacidopathies and organic acidurias tolerated COVID-19 relatively well. Disorders such as organic acidurias did not inevitably result in severe COVID-19 and most affected patients had only mild symptoms [5]. However, some studies with a higher proportion of decompensation-prone patients (e.g., UCD and organic acidurias) reported slightly higher rates of severe infection [4]. In our cohort, several patients with propionic, methylmalonic, or isovaleric aciduria contracted COVID-19, with episodes of metabolic imbalance in two cases of propionic aciduria, but without requiring hospitalization.

In published studies, only a very small fraction of patients required ICU care for hyperammonemia or escalation of therapy, generally in the context of severe COVID-19 pneumonia.

#### Mitochondrial disorders

Patients with primary mitochondrial diseases (including respiratory chain defects, pyruvate metabolism disorders, etc.) may have significant neuromuscular and cardiac involvement, which raises the theoretical concern that a systemic infection like COVID-19 could trigger a severe energy crisis or organ failure. In our study, some patients with mitochondrial disease required hospitalization, including one child admitted to the ICU.

International data are variable. Some reports describe poor COVID-19 outcomes: an international, cross-sectional, registry-based study including 79 patients found that 32% were hospitalized and 4% died (respiratory dysfunction was a risk factor for hospitalization) [27].

In contrast, broader analyses have not found a correlation between having an energy metabolism disorder and a more severe COVID-19 prognosis [4]. Interestingly, certain metabolic diseases like lipin1 deficiency may protect against COVID-19 due to membrane alterations that prevent viral entry [28], and our patients with this deficiency did not get sick. Of course, supportive measures remain essential, for example ensuring adequate hydration and nutrition during infection, and maintaining any metabolic supplementation to prevent decompensation (e.g., ketogenic diet in pyruvate-related disorders). The reassuring outcomes observed should be interpreted with caution given the small number of cases, but overall, current data do not indicate that patients with mitochondrial disease experience disproportionate COVID-19 severity.

**Glycogen storage disorders (GSDs)** include a range of conditions (types I, III, V, etc.) with varying impacts on the liver, muscles, and heart. The main acute risk in many GSDs, especially type I, is hypoglycemia.

In the series of Altassan R et al., patients with GSD I and GSD III who contracted COVID-19 did not experience severe decompensation and did not require hospitalization [4].

In our cohort, the impact of COVID-19 on the disease was significant, with one child admitted to the ICU for metabolic decompensation and some adults requiring hospitalization, despite caregivers well trained to intensify cornstarch therapy or enteral feeding at the first signs of reduced intake or to opt for hospital care.

No cases of liver failure or serious myopathic complications were observed in our GSD patients.

**Aside from emergency protocols**, which, if applied promptly during infection, help prevent decompensation and are routinely taught to patients as part of therapeutic education [10], other evolving factors such as vaccination have significantly changed the landscape. Most of our data were collected before widespread vaccine availability, whereas now, the majority of IMD patients are vaccinated, and booster doses are prioritized for them. Recent studies have shown that vaccination has further reduced the risk of both severe COVID-19 and IMD decompensation in these patients [29].

### Limitations

We acknowledge several limitations in our study. First, the study relies on voluntary reporting from reference centers, which may introduce selection bias, particularly favoring report of symptomatic cases and leading to underreporting of mild or asymptomatic COVID-19 cases among IMD patients. Second, our study does not provide an exhaustive account of all COVID-19 infections in patients with IMDs in France, as participation was based on voluntary reporting from expert centers. Indeed, the IMD patient cohort in 2021—defined as patients with a confirmed diagnosis of an IMD who were evaluated at least once during the year by a rare disease expert center— included 9,560 individual patients (4,558 children [< 18 years] and 5,002 adults) in the French National Rare Diseases Data Bank (BNDMR). Thirdly, no inter-center validation nor centralized case review was performed. Finally, our follow-up was limited to the course of the acute infection, and we did not systematically assess long-term effects such as post-COVID syndrome or long-COVID effects or the impact of prolonged healthcare delays on metabolic control.

## Conclusions

This large French cohort of 317 patients with IMDs infected with COVID-19 shows that the reported infection was mostly mild to moderate. Children had a higher risk of metabolic destabilization and ICU admission than adults, particularly in cases of energy-related metabolic disorders. However, no death occurred in children with IMDs. Conversely, one death occurred in an adult patient with Fabry disease, and all four adult patients admitted to the ICU had LDs. Treatment suspension or delay was rare.

This overall favorable outcome of COVID-19 infection within the French IMDs Healthcare Network for Rare Diseases suggests that, while the risk is real, it may be lower than initially feared if preventive measures are put in place. Indeed, this study also highlights the importance of coordinating care, preventing metabolic decompensation (emergency protocols), and anticipating potential treatment interruptions during epidemics or pandemics. Finally, this overall good prognosis also raises the hypothesis that the pathophysiology of some IMDs could modulate the risk of COVID-19 infection and severity.

## Supplementary Information

Below is the link to the electronic supplementary material.

Supplementary Material 1

Supplementary Material 2

## Data Availability

The datasets used and/or analysed during the current study are available from the corresponding author on reasonable request.

## Abbreviations

AA: Aminoacidopathies
ACE2: angiotensin-converting enzyme2
AIP: Acute intermittent porphyria
ASMD: Acid sphingomyelinase deficiency
CPS1: Carbamylphosphate Synthase 1
FAOD: Fatty acid oxidation disorders
GSD: Glycogen storage disease
ICU: Intensive care unit
IMD: Inherited metabolic disease
LDs: Lysosomal disorders
MADD: Multiple acyl-CoA dehydrogenase deficiency
MELAS: Mitochondrial Encephalomyopathy Lactic Acidosis Stroke-like episodes
MPS: Mucopolysaccharidoses
MSUD: Maple syrup urine disease
MTPD: Mitochondrial trifunctional protein deficiency
PDHD: Pyruvate dehydrogenase deficiency
PKU: Phenylketonuria
PMM2-CDG: Phosphomannomutase deficiency - Congenital disorders of glycosylation
Pompe: Pompe disease
UCD: Urea cycle disorders
VLCAD: Very long-chain acyl-CoA dehydrogenase deficiency
Wilson: Wilson disease
