# Supplementary material for: Impact of COVID-19 infection in patients with inherited metabolic diseases: a National Multicenter Study from the French IMDs Healthcare Network for Rare Diseases
Source: Orphanet J Rare Dis. 2026 Feb 14;21:71. doi: 10.1186/s13023-026-04230-8 (PMC12922317; doi:10.1186/s13023-026-04230-8)
Supplement: Supplementary file 1 — Supplementary Material 1 [file 13023_2026_4230_MOESM1_ESM.docx]

**Supplemental Data 1. Biochemical criteria used to define metabolic decompensation**

- **Pyruvate dehydrogenase deficiency:** lactic acidosis (pH < 7.35, anion gap > 20), elevated blood lactate levels, and increased pyruvate.
- **Urea cycle disorders due to carbamyl phosphate synthetase 1 (CPS1) deficiency or ornithine transcarbamylase (OTC) deficiency:** elevation of plasma ammonia above the laboratory upper limit of normal, or above the patient’s usual baseline if ammonia is chronically borderline). Plasma amino acid chromatography subsequently provides additional information, including elevated glutamine (> 800 µmol/L), decreased citrulline and arginine, and increased urinary orotic acid in OTC deficiency (but low urinary orotic acid in CPS1 deficiency).
- **Glycogen storage disease type I:** hypoglycemia [blood glucose ≤ 0.6 g/L (3.3 mmol/L)], lactate > 2.8 mmol/L, bicarbonate < 20 mmol/L, triglycerides > 5.7 mmol/L (5 g/L), and serum uric acid above the upper limit of normal for age and laboratory reference ranges.
- **Glycogen storage disease type III:** hypoglycemia [blood glucose ≤ 0.6 g/L (3.3 mmol/L)], increase in creatine kinase (CK) above the usual level of the patient, increased lactate and transaminase levels.
- **Glycogen storage disease type V:** increase in CK above the usual level of the patient
- **Mitochondrial trifunctional protein deficiency (MTPD), multiple acyl-CoA dehydrogenase deficiency (MADD), and very long-chain acyl-CoA dehydrogenase deficiency (VLCAD):** hypoglycemia [blood glucose ≤ 0.6 g/L (3.3 mmol/L)], absence of ketone bodies, elevated CK, increased lactate and plasma ammonia levels, and occasionally acute hepatocellular failure.
- **Propionic acidemia:** lactic acidosis (pH < 7.35, anion gap > 20), with increased lactate and plasma ammonia levels.
- **Maple syrup urine disease (MSUD):** metabolic decompensation defined by leucine > 400 µmol/L (severity criterion if > 800 µmol/L), presence of alloisoleucine (> 5 µmol/L), and moderate elevation of valine (> 300 µmol/L) and isoleucine (> 80 µmol/L).
- **Wilson disease:** worsening hepatocellular injury and hyperbilirubinemia
