# Supplementary material for: Impact of COVID-19 infection in patients with inherited metabolic diseases: a National Multicenter Study from the French IMDs Healthcare Network for Rare Diseases
Source: Orphanet J Rare Dis. 2026 Feb 14;21:71. doi: 10.1186/s13023-026-04230-8 (PMC12922317; doi:10.1186/s13023-026-04230-8)
Supplement: Supplementary file 2 — Supplementary Material 2 [file 13023_2026_4230_MOESM2_ESM.docx]

**Supplemental Table 1. Number of patients hospitalized or in ICU in 2020 (before vaccination) and 2021-2022 (after vaccination)**

|  | Missing date of COVID | Number of patients hospitalized or in ICU in 2020 | Number of patients hospitalized or in ICU in 2021 or 2022 |
| --- | --- | --- | --- |
| children | 22 | 2/4 (50%) | 8/43 (19%) |
| adults | 17 | 7/46 (15%) | 10/185 (5%) |
